# Retrograde Snaring for Left Ventricular Lead Placement in the Presence of a Persistent Left Superior Vena Cava

**DOI:** 10.19102/icrm.2023.14012

**Published:** 2023-01-15

**Authors:** Enes E. Gul, Ibrahim Ahmad Ali, Yumna B. Haseeb, Sohaib Haseeb, Osama Al Amoudi

**Affiliations:** ^1^Division of Cardiac Electrophysiology, Madinah Cardiac Center, Madinah, Saudi Arabia; ^2^Department of Biomedical and Molecular Sciences, Queen’s University, Kingston, Ontario, Canada; ^3^College of Medicine and Dentistry, James Cook University, Townsville, Queensland, Australia

**Keywords:** Cardiac resynchronization therapy, left ventricular lead, persistent left superior vena cava, snare technique

## Abstract

Left ventricular lead positioning is technically demanding in cardiac resynchronization therapy (CRT) device implantation, especially in patients with complex cardiac venous anatomies. We report a case in which retrograde snaring was employed to successfully deliver the left ventricular lead through a persistent left superior vena cava for CRT implantation.

## Introduction

Successful cardiac resynchronization therapy (CRT) requires precise positioning of the left ventricular (LV) lead.^1^ Complex and otherwise variable cardiac venous anatomy can often impede successful delivery of the LV lead to its optimal target site.^2^ In this context, a persistent left superior vena cava (PLSVC) is the most common thoracic venous anomaly, which mostly drains into the right atrium (RA) through the coronary sinus (CS).^3^ Although asymptomatic, it can make LV lead implantation more challenging and can cause incorrect positioning, dislodgement, or failure.^4^ Consequently, approaches to navigate narrow, tortuous, and complex anatomy have been described.^5–8^ We describe a case in which retrograde snaring was employed to successfully deliver the LV lead through a PLSVC for CRT implantation.

## Case presentation

A 54-year-old man with a medical history of dilated cardiomyopathy was admitted electively for CRT implantation. On admission, his functional capacity corresponded to New York Heart Association class III despite optimal medical treatment for heart failure. His electrocardiogram (ECG) showed sinus rhythm with typical left bundle branch block and an underlying QRS duration of 152 ms. A transthoracic echocardiogram detected impaired LV systolic function with an ejection fraction (EF) of 25% and moderate functional mitral regurgitation. Given the available clinical, ECG, and imaging findings at the time, CRT implantation was pursued.

**Figure 1:**
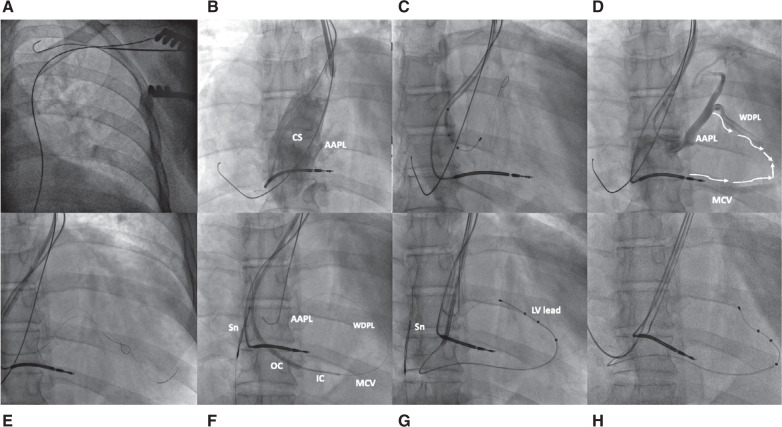
Stepwise fluoroscopic imaging in the left anterior oblique view showing successful implantation of a cardiac resynchronization therapy defibrillator in a patient with a persistent left superior vena cava. **A:** Guidewires in the persistent left superior vena cava. **B:** Cororonary sinus angiogram showing a large coronary sinus with a small acute angulated posterolateral branch. **C:** Attempted antegrade placement of the left ventricular lead into the branch. **D:** Selective angiogram showing a well-developed posterolateral branch with collateral connection (white arrows) toward the middle cardiac vein. **E:** Hi-Torque Whisper™ and Pilot™ guidewires (Abbott) inside the posterolateral branch. **F:** Selective engagement of the middle cardiac vein with both outer and inner catheters and the guidewire advanced to the right atrium via the collaterals and snared in the right atrium. **G:** Left ventricular lead advanced over the snared guidewire. **H:** Final lead positions. *Abbreviations:* AAPL, acute angulated posterolateral branch; CS, coronary sinus; IC, inner catheter; LV, left ventricular; MCV, middle cardiac vein; OC, outer catheter; PLSVC, persistent left superior vena cava; Sn, snare; WDPL, well-developed posterolateral branch.

The patient was admitted to the cardiology unit for CRT defibrillator (CRT-D) implantation. Venography confirmed a PLSVC with no antegrade flow into the SVC **(Figure 1A)**. It was decided to proceed with device implantation via the PLSVC. The right ventricular (RV) lead was properly positioned in the RV apex and the RA lead was positioned in the lateral atrial wall, with satisfactory parameters. We introduced a CS delivery outer guide catheter (CPS Direct SL II 115; Abbott, Chicago, IL, USA), and contrast injection inside the CS revealed a posterolateral branch with acute angulation **(Figure 1B)**. This branch was selectively cannulated with a subselective inner catheter (CPS AIM Universal; Abbott), and an angiogram revealed a relatively well-developed posterolateral branch (WDPL) with collaterals toward the middle cardiac vein (MCV) **(Figures 1C and 1D)**. Despite several attempts, the LV lead could not be advanced due to an unfavorable angle **(Figure 1E)**. Subsequently, the MCV with well-developed collaterals was engaged with a vertebral vein selector sheath (Merit Medical, South Jordan, UT, USA) toward the posterolateral branch **(Figure 1F)**. However, the LV lead could not be negotiated to an optimal position. Finally, the guidewire was advanced into the RA through the collaterals (initially WDPL, then acutely angulated posterolateral branch) via the posterolateral branch, and the distal end of the wire was left in the high RA **(Figure 1G)**. A snare (Trefoil ensnare/TriSnare; Merit Medical) was introduced through the right femoral vein, and the guidewire was captured in the RA to create a venovenous loop. The LV lead was tracked into its desired position with the aid of a veno-venous loop **(Figure 1H)**. Afterward, the guidewire was removed through the femoral path **(Video 1)**. Pacing and sensing thresholds were satisfactory, without diaphragmatic stimulation. No dislodgement of the lead was observed after slitting the CS delivery sheath and the subselective guide catheter.

Thus, all 3 leads were implanted through the PLSVC. The total procedure duration and fluoroscopy time were 140 and 34 min, respectively. There were no complications.

Six months following the procedure, all lead parameters were satisfactory, and a repeat echocardiogram revealed slight improvements in the patient’s functional capacity (LV ejection fraction, 35%).

## Discussion

Several maneuvers have been described to facilitate LV lead placement to overcome unfavorable cardiac venous anatomy, including the use of (1) single or multiple buddy wires,^9^ (2) subselective guide catheters or microcatheters,^10^ (3) an anchor wire,^11^ and (4) venoplasty.^12^ Collateral branches have also been used in patients with a CS obstruction.^5,6^ To this end, retrograde access of the anterolateral CS branch using collaterals and externalization through the CS ostium has also been reported.^13^

The use of snaring to assist with the placement of the LV lead has also demonstrated its success. The “Worley loop technique” is one such method that involves a standard gooseneck snare and a venovenous loop. In brief, the snare is introduced into the CS through the same catheter as the lead delivery guidewire and the other end of the guidewire is held in the CS.^14^ There are several instances where LV lead implantation has been successful through this technique. In a case of CRT implantation, a snare system was employed inside the RA to capture the end of the guidewire, forming a loop to advance the LV lead into its desired position.^15^ In another instance, a guidewire was snared from the SVC and a venovenous loop was formed to aid the advancement of the LV lead over a tortuous CS segment in CRT-D implantation.^10^ A modification to the Worley snare technique—in which a secondary CS sheath is introduced to deliver the snare—was shown to improve lead traction and promote LV lead delivery in the event of narrow or tortuous vascular anomalies.^16^

Utilization of the snare technique in patients undergoing LV lead placement in the presence of a PLSVC has not been widely reported.^17^ Our case lends further support to the use of this approach to assist in the placement of the LV lead in patients with anatomical variants, such as a PLSVC.

## Conclusion

In patients with a PLSVC undergoing CRT therapy, both antegrade and retrograde snaring can be utilized to negotiate the LV lead to a desired and stable position. Greater experience will allow operators to establish proficiency with this technique.

## Figures and Tables

**Video 1:** Successful implantation of a cardiac resynchronization therapy defibrillator in a patient with a persistent left superior vena cava.
